# *N*^6^-Methyladenosine modification (m^6^A) of circRNA-ZNF638 contributes to the induced activation of SHF stem cells through miR-361-5p/Wnt5a axis in cashmere goats

**DOI:** 10.5713/ab.22.0211

**Published:** 2022-11-14

**Authors:** Ronghuan Yin, Ronglan Yin, Man Bai, Yixing Fan, Zeying Wang, Yubo Zhu, Qi Zhang, Taiyu Hui, Jincheng Shen, Siyu Feng, Wenlin Bai

**Affiliations:** 1 College of Animal Science and Veterinary Medicine, Shenyang Agricultural University, Shenyang 110866, China; 2 Research Academy of Animal Husbandry and Veterinary Medicine Sciences of Jilin Province, Changchun, 130062, China

**Keywords:** Cashmere Goats, CircRNA-ZNF638, MiR-361-5p, *N*^6^-Methyladenosine, SHF Stem Cells, Wnt5a

## Abstract

**Objective:**

The objective of this study was to investigate the effects of *N*^6^-Methyladenosine modification-circRNA-zinc finger protein 638 (m^6^A-circRNA-ZNF638) on the induced activation of secondary hair follicle (SHF) stem cells with its potential mechanisms in cashmere goats.

**Methods:**

The m^6^A modification of ZNF638 was analyzed using methylation immunoprecipitation with real-time quantitative polymerase chain reaction technique in SHF stem cells. The effects of circRNA-ZNF638 on the induced activation of SHF stem cells in m^6^A dependence were evaluated through the overexpression of circRNA-ZNF638/its m^6^A-deficient mutants in circRNA-ZNF638 knockdown SHF stem cells. The competitive binding of miR-361-5p to circRNA-ZNF638/Wnt5a 3′-untranslated region was analyzed through Dual-luciferase reporter assay.

**Results:**

The m^6^A-circRNA-ZNF638 had significantly higher transcription at anagen SHF bulge of cashmere goats compared with that at telogen, as well as it positively regulated the induced activation of SHF-stem cells in cashmere goats. Mechanismly, m^6^A-circRNA-ZNF638 sponged miR-361-5p to heighten the transcriptional expression of *Wnt5a* gene in SHF-stem cells. We further demonstrated that the internal m^6^A modification within circRNA-ZNF638 is required for mediating the miR-361-5p/Wnt5a pathway to regulate the induced activation of SHF stem cells through an introducing of m^6^A-deficient mutant of circRNA-ZNF638.

**Conclusion:**

The circRNA-ZNF638 contributes the proper induced activation of SHF-stem cells in cashmere goats in m^6^A-dependent manner through miR-361-5p/Wnt5a axis.

## INTRODUCTION

Cashmere is a precious natural protein fiber that derives from the secondary hair follicles (SHFs) of cashmere goats, and it has been regarded as high-grade textile raw material [1]. As one of the main products from cashmere goats, cashmere has important economic implications to local farmers and herdsmen. The morphogenesis and growth of cashmere is controlled by the activity of SHFs in cashmere goats [2]. Under the dermal papilla cell (DPC)-derived signals, the induced activation of SHF stem cells is significantly important for the reconstruction of SHFs with the subsequent the morphogenesis and growth of cashmere fibers in cashmere goats [3]. It is known that the induced activation of hair follicle stem cells is coordinately regulated by a variety of endogenous regulatory factors [4]. There is evidence that the Wnt/β-catenin signals can induce the activation of hair follicle stem cells [5]. There are also other signals and molecules implicated in the induced activation process of hair follicle stem cells, such as mammalian target of rapamycin [6], tumor necrosis factor [7], platelet-derived growth factor subunit A [8], and Foxi3 [5]. Also, it was reported that some miRNAs were also implicated in the activation event of hair follicle stem cells [4], such as miR-339-5p [9] and miR-214 [10].

*N*^6^-methyladenosine (m^6^A) has been revealed to be the most abundant modification in linear RNA molecules. Over the past few years, extensive m^6^A modifications sites are also found in many circRNA molecules that play significant roles in a variety of biological cells through m^6^A modification dependent model [11]. To date, a considerable number circRNAs have been identified in skin tissue or SHFs of cashmere goat [12], and some of them are demonstrated to positively regulate the differentiation of SHF stem cells into hair follicle lineage, such as circRNA-1926 [13], circRNA-1967 [14], and circRNA-0100 [15]. Moreover, some circRNAs in SHFs of cashmere goats are verified to contain multiple m^6^A modification sites [2]. Increasing lines of evidence indicate that the m^6^A modifications are heavily implicated in the functional exertion of the m^6^A modified circRNAs (m^6^A-circRNAs) via m^6^A-dependent model [11, 16].

In our recent study, a novel m^6^A-circRNA, named as m^6^A-circRNA-ZNF638, was identified in SHFs of cashmere goats, and it was transcribed from the antisense strand of goat zinc finger protein 638 (*ZNF638*) gene with significantly higher expression at anagen SHFs than that at telogen [2]. As a matter of fact, the ZNF638 is a member of ZNF family. Although the functional roles of ZNF638 in SHFs of cashmere goat remains unclear, several members of this family (ZNFs) were demonstrated to play significant roles in the physiological processes of cashmere goat SHFs including g ZNF264, ZNF347, ZNF407, ZNF454, ZNF667, and ZNF704 [17]. Considering that the SHF stem cells are under the vigorous status of induced activation at anagen, we hypothesize that the m^6^A-circRNA-ZNF638, as a circular RNA molecule transcribed from the antisense strand of *ZNF638* gene, may be implicated in the induced activation process of SHF stem cells of cashmere goats.

In this study, we firstly assessed the relative expression of m^6^A-circRNA-ZNF638 in SHF bulge of cashmere goats at anagen and telogen. Further, we evaluated the putative effects of m^6^A-circRNA-ZNF638 on the induced activation of SHF stem cells of cashmere goats and investigated its possible functional mechanisms. Our results provided new molecular evidence for unveiling the potential molecular mechanisms of the induced activation event of SHF stem cells in cashmere goats.

## MATERIALS AND METHODS

### Sample collection, preparation, and total RNA isolation

In this study, all experimental protocols were reviewed and approved by the Experimental Animal Ethics and Welfare Committee of Shenyang Agricultural University (Shenyang, China) under the ethical code: 201606005. Correspondingly, the experiments were performed based on the approved procedure guidelines. The skin tissues (body side) were collected from nine cashmere goats (Liaoning cashmere breed, female, three-year-old, no traceable common genetic relationships) as described in our previous investigation [14]. In brief, the skin tissues were sampled from each goat using a sterilized surgical blade. The collected skin samples were cleaned with 75% alcohol. Subsequently, the skin samples were cut into pieces of 5 mm^2^ and washed with PBS for 3 times. The samples were further digested at 4°C with dispase II (0.25%; Roche, Mannheim, Germany) for 8 h. Under a stereo microscope, the SHFs were isolated with a sterilized scalpel and disposable syringe needle, and the SHF bulge sections were isolated following described protocol by Ohyama and Kobayashi [18]. The total RNA was extracted from SHF bulge using RNAiso reagent kit (TaKaRa, Dalian, China) according to the manufacturer’s recommendations.

### Sequence of m^6^A-circRNA-ZNF638 and its in-silico analysis

The analyzed m^6^A-circRNA-ZNF638 has been identified from SHFs of cashmere goats in our previous study [2]. The sequence of m^6^A-circRNA-ZNF638 was managed and displayed with BioEdit software [19]. We determined the transcribed source of m^6^A-circRNA-ZNF638 in goat genome through aligning its linearized sequence into the goat genome (https://www.ncbi.nlm.nih.gov/genome/?term= goat, assembly, ARS1, last access: 18 March 2022). The potential target binding miRNAs within m^6^A-circRNA-ZNF638 sequence were predicted using two procedures (RNAhybrid: https://bibiserv.cebitec.uni-bielefeld.de/rnahybrid, and miRDB: http://www.mirdb.org, last access: 18 March 2022), and were further selected among the resulting miRNAs through taking an intersection. The goat miRNA sequences were retrieved from the miRNA database: miRNAsong (https://www2.med.muni.cz/histology/miRNAsong/index.php, last access: 18 March 2022). The potential m^6^A sites within circRNA-ZNF638 sequence were screened using the SRAMP procedure (http://www.cuilab.cn/sramp, accessed on 11 July 2021).

### Overexpression/siRNA interference and cell co-cultivation

The SHF-stem cells (stored in our laboratory) were used in the overexpression/siRNA interference analysis of m^6^A-circRNA-ZNF638. The overexpression analysis of circRNA-ZNF638 (or its mutant) was performed in SHF-stem cells of cashmere goats, where the mutations of circRNA-ZNF638 were generated using the QuikChange Lightning Multi Site-Directed Mutagenesis Kit (Agilent, Technologies, Santa Clara, CA, USA) following the manufacturer’s recommendations. Overexpression vectors were constructed as described protocol in previous publication [13]. In brief, the pcDNA3.1 (+) circRNA mini-vector (Addgene, Cambridge, MA, USA) was utilized in overexpression assay. The SHF stem cells were transiently transfected with the recombinant pcDNA3.1 (+) circRNA-ZNF638 (or its mutant), which was carried out by the Lipofectamine 3000 (Invitrogen, Carlsbad, CA, USA). For the knockdown assay of circRNA-ZNF638, we designed three specific siRNAs (si-circR-1, si-circR-3, and si-circR-3) that potentially targeted the back splice region of circRNA-ZNF638. The three siRNA sequences are si-circR-1: 5′-AT GTAGTCATTTGAACTTTGTG-3′, si-circR-2: 5′-GAATG TAGTCATTTGAACTTTG -3′, and si-circR-3: si-circR-3: 5-TCATTTGAACTTTGTGTTATTC-3′. These siRNAs were synthesized commercially by GenePharma Co., Ltd. (Shanghai, China). Using the siRNAs Lipofectamine RNAiMAX kits (Invitrogen, Shanghai, China), the siRNAs were transiently transfected into SHF stem cells according to the manufacturer’s recommendations.

The transfected SHF stem cells were co-cultured with DPCs (stored in our laboratory) for induced activation in a transwell device as described by Yan and colleagues [4]. In brief, the DPCs (passage 3) of cashmere goats were seeded on a transwell insert, which was added to a six-well plate seeded with SHF stem cells (passage 3) of cashmere goats. Subsequently, in fresh DMEM/F12 medium (Hyclone, Logan, UT, USA; supplemented with 10% fetal bovine serum), the cells were non-contactingly co-cultured under a humidified atmosphere at 37°C with 5% CO_2_. The culture media was replaced every two days. After post-transfection 48 h, the transfected cells were collected, and the transfection efficiency was evaluated by RT-qPCR assay. The transfection efficiency was above 70%.

### Primer designing and RT-qPCR assay

For m^6^A-circRNA-ZNF638, divergent primer pair was cited from the previous study [2], and for the methylation immunoprecipitation (Me-RIP) with real-time quantitative polymerase chain reaction (RT-qPCR) of m^6^A-circRNA-ZNF638 and mRNAs of the related genes, convergent primer pairs were designed using the Primer Premier 5.0 program (http://www.premierbiosoft.com). Whereas, for miRNAs, the sense primer of each miRNA was designed based on its mature sequence that was retrieved from the miRNA database: miRNAsong (https://www2.med.muni.cz/histology/miRNAsong/index.php), and the anti-sense primers are universal to all miRNAs that were obtained from the kits (TaKaRa, China). The primer pair of U6 was cited from the previous study [20]. The details of all primers are presented in Table 1.

For the expression analysis of m^6^A-circRNA-ZNF638 (or it’s Me-RIP-qPCR) and mRNAs of implicated genes, the M-MuLV cDNA synthesis kit (Sangon, Shanghai, China) was used for synthesizing the first strand cDNA with random primers. Whereas for miRNA expression analysis, the One-Step PrimeScript microRNA cDNA synthesis kit (TaKaRa, China) was used for synthesizing the first strand cDNAs. The RT-qPCR assay carried out in a light Cycler 480 real-time PCR system (Roche Diagnostics, Germany). In a 25 μL final volume, RT-qPCR amplication was performed using TB Green Premix Ex Taq II (Tli RNaseH Plus; TaKaRa, Chnia) containing 1.0 μL each primer (10 μM), 2.0 μL first-strand cDNA solution, 12.5 μL TB Green Premix Ex Taq II (Tli RNaseH Plus; TaKaRa, Chnia), and 8.5 μL PCR-grade ddH_2_O water. We used the following thermal cycling parameters: a single cycle (95°C for 30 s) followed by 40 cycles: 95°C for 5 s, 53°C to 60°C (Table 1) for 30 s, and 72°C for 30 s. The m^6^A relative enrichment of m^6^A-circRNA-ZNF638 was normalized to the input, and the mRNA relative expression of the implicated genes was calculated with the 2^−ΔΔCt^ method.

### Methylation Immunoprecipitation of m^6^A-circRNA-ZNF638 (Me-RIP) in SHF stem cells

The Me-RIP analysis of m^6^A-circRNA-ZNF638 was conducted as described protocol by Chen and colleagues [21]. Briefly, 100 μg total RNA was treated with RNase R (Geneseed, Guangzhou, China), and further concentrated using Monarch RNA Cleanup Kit (NEB, Ipswich, MA, USA). Subsequently, RNA was fragmented at 94°C for 3 min using the NEBNext Magnesium RNA Fragmentation Module (NEB, USA), and further concentrated with the Monarch RNA Cleanup Kit (NEB, USA). The fragmented product of 2 μg was reserved as input control. Half fragmented RNA was incubated anti-m^6^A antibody of 2 μg (Synaptic Systems, Gottingen, Germany) or IgG of 2 μg (Cell Signaling Technology, Danvers, MA, USA) at 4°C for 4 h. After pre-washing, the Dynabeads Protein A (Thermo Scientific, Rockford, IL, USA) was subjected to incubation with the complex of RNA and antibody at 4°C for 2 h. The RNA was extracted with the RNAiso kit (TaKaRa, China), and the expression of m^6^A-circRNA-ZNF638 of each sample was analyzed via the RT-qPCR assay.

### Dual-luciferase reporter assays

The dual-luciferase reporter assay was carried out as described in previous publication [22]. In brief, Luciferase reporters were generated by cloning circRNA-ZNF638 (circRNA-ZNF638-WT) or its mutant (circRNA-ZNF638-MUT) into pGL3-basic vectors (Promega, Madison, WI, USA). The mutant of circRNA-ZNF638 (circRNA-ZNF638-MUT), that was mutated within the potential binding sites of miR-361-5p seed region, were generated using the QuikChange Lightning Multi Site-Directed Mutagenesis Kit (Agilent, Technologies, USA) following the manufacturer’s recommendations. Also, the 3′-untranslated region (3′-UTR) fragment of cashmere goat Wnt5a mRNA was amplified, which contained the potential binding site of miR-361-5p and was ligated to the pGL3 basic vector (Promega, USA). And then, the SHF-stem cells (passage 3) of cashmere goats were prepared and transfected with the generated reporter vectors via the use of Lipofectamine 2000 (Invitrogen, USA). After post-transfection 48 h, the transfected cells were collected, and the transfection efficiency was evaluated by RT-qPCR assay. The transfection efficiency is above 70%. The transfected cells were cultured under the above-mentioned conditions. After 48 h, the cell lysates were harvested and luciferase activities were examined using the Dual-Luciferase Reporter Assay System (Promega, USA). The ratio of firefly to renilla luciferase activity was measured to eliminate possible bias in transfection efficiency.

### Data statistical analysis

Statistical analysis of obtained data was performed with the SPSS 17.0 program (SPSS Inc., Chicago, IL, USA). The data was presented as mean±standard deviation. The comparison for potential difference between two groups was performed with the Student’s *t*-test. The resulting p-values less than 0.05 were considered statistically significant.

## RESULTS AND DISCUSSION

### Molecular characterization of m^6^A-circRNA-ZNF638 in SHFs of cashmere goats

**Figure 1:**
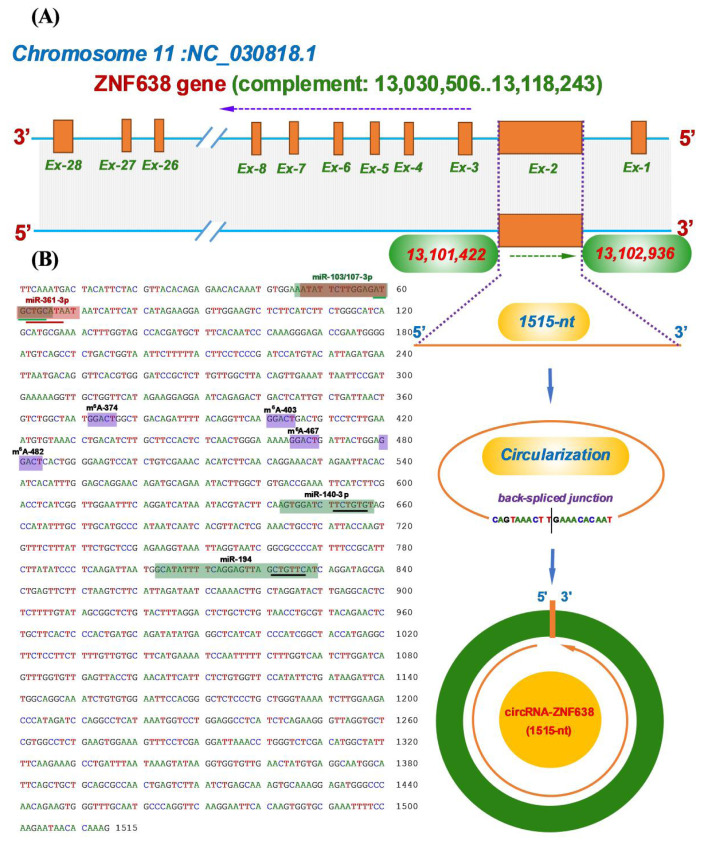
The reverse splicing source of circRNA-ZNF638 in cashmere goats with its molecular characteristics. (A) Overall diagram of the host source of circRNA-ZNF638 and its reverse splicing model with the size of 1515-nt. (B) Display of a circRNA-ZNF638 cDNA sequence. The circRNA-ZNF638 cDNA harbors the potential binding sites of five miRNAs indicated by shading with the respective miRNA name where the potential binding region of seed sequence of each miRNA was indicated by underline. Also, it contains four m^6^A modification sites indicated by shading with the same motif of GGACU including m^6^A-374, m^6^A-403, m^6^A-467, and m^6^A-482. circRNA-ZNF638, circRNA-zinc finger protein 638.

Here, the analyzed m^6^A-circRNA-ZNF638 has been identified from cashmere goat SHFs in our previous study [2]. Through an alignment of its linearized sequence into the goat genome (https://www.ncbi.nlm.nih.gov/genome/?term=goat,assembly,ARS1), it was revealed that the m^6^A-circRNA-ZNF638 was transcribed from the antisense strand of goat *ZNF638* gene (Figure 1A). In fact, the goat *ZNF638* gene contains multiple exons including exons 1 to 28, which was well annotated in NCBI database (https://www.ncbi.nlm.nih.gov). In detail, the m^6^A-circRNA-ZNF638 is formed via a reverse splicing of the antisense strand of entire exon 2 of the goat *ZNF638* gene with position nos. 13101422–12102936 of the NC_030818.1 sequence on chromosome 11 (Figure 1A), and it is 1515-nt in spliced length.

Through in-silicon analysis, we also noted that m^6^A-circRNA-ZNF638 contained potential target sites of multiple miRNAs, including miR-103-3p, miR-107-3p, miR-361-5p, miR-140-3p, and miR-194 (Figure 1B), which implies that m^6^A-circRNA-ZNF638 in SHFs of cashmere goats may exert its biological functions through miRNA-mediated pathways as reported in recent publications [13–15]. On the other hand, the four m^6^A sites within m^6^A-circRNA-ZNF638 sequence were revealed to have the same motif of GGACU, which is consistent with the m^6^A motif: RRACH (R, A/G; H, A/C/U) reported in linear RNA molecules (Figure 1B) [23].

### Expression characterization of m^6^A-circRNA-ZNF638 in SHF bulge of cashmere goats and its effects on the induced activation of SHF-stem cells

**Figure 2:**
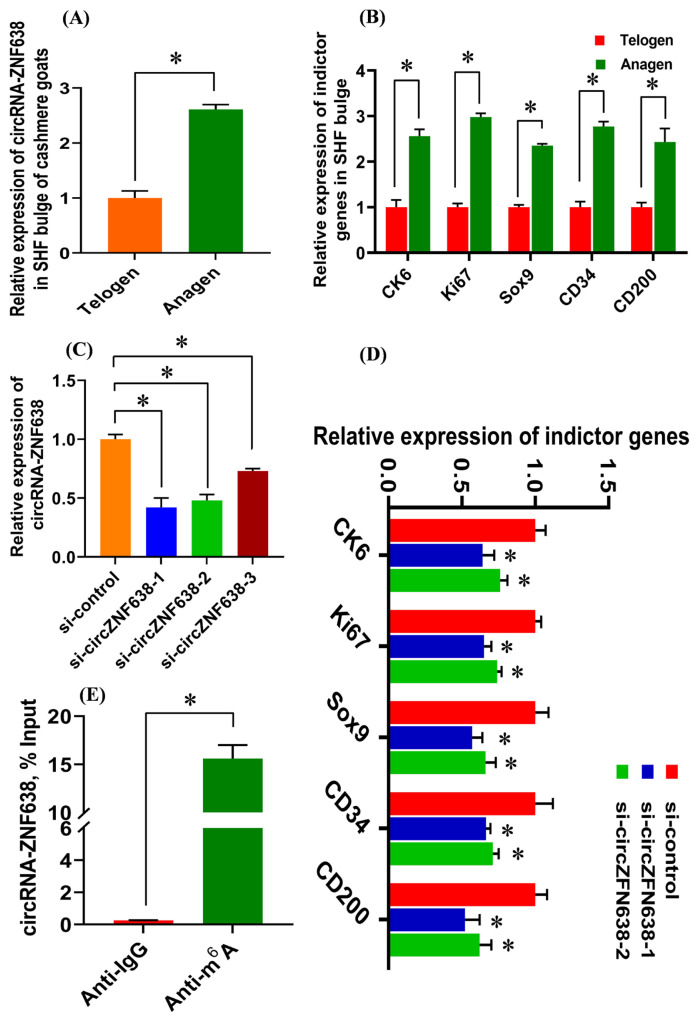
The expression analysis of m^6^A-circRNA-ZNF638 in the SHF bugle of cashmere goats and the effects of m^6^A-circRNA-ZNF638 on the induced activation of SHF-stem cells in cashmere goats. (A) Expression pattern of m^6^A-circRNA-ZNF638 in SHF bugle of cashmere goats. (B) Expression pattern of indicator genes on the induced activation of SHF-stem cells in SHF bugle of cashmere goats. (C) Knockdown efficiency analysis of si-circZNF638-1: 5′-ATGTAGTCATTTGAACTTTGTG-3′, si-circZNF638-2: 5′-GAATGTAGTCATTTGAACTTTG -3′, and si-circZNF638-3: 5-TCATTTGAACTTTGTGTTATTC-3′ to m^6^A-circRNA-ZNF638 in SHF-SCs of cashmere goats. (D) Knockdown of m^6^A-circRNA-ZNF638 led to the significant decrease in expression level of the analyzed indictor genes in SHF-stem cells. (E) Enrichment of m6A-modified circRNA-ZNF638 in SHF-stem cells of cashmere goats. The percentage of the input is shown. m^6^A-circRNA-ZNF638, *N*^6^-Methyladenosine modification-circRNA-zinc finger protein 638; SHF, secondary hair follicle. The asterisk “*” indicates significant difference (p<0.05).

For revealing the expression characterization of m^6^A-circRNA-ZNF638 in the SHF bulge of cashmere goats, two main phases of SHF cycle were investigated including anagen and telogen [1]. As observed from Figure 2A, m^6^A-circRNA-ZNF638 exhibited significantly higher expression in the anagen SHF bulge compared with that of telogen. As well known, at anagen bulge of cashmere goats, the SHF-stem cells are under vigorous status of induced activation from the DPC signal stimulation to drive the regeneration and growth of SHFs [3]. As observed from Figure 2B, this was also supported well by the tested results from this study, where several indicator genes upon induced activation of SHF-stem cells exhibited significantly higher expression at anagen bulge compared with those at telogen including keratins CK6, Ki67, Sox9, CD34, and CD200 [5,24,25]. Thus, we speculate that m^6^A-circRNA-ZNF638 may be significantly implicated in the induced activation event of SHF-stem cells in cashmere goats.

To confirm this hypothesis, we carried out the knockdown experiment of m^6^A-circRNA-ZNF638 in SHF-stem cells of cashmere goats through introducing siRNA inference molecules. Three independent siRNAs were designed including si-circZNF638-1, si-circ ZNF638-2, and si-circ ZNF638-3, and their knockdown efficiencies were verified in SHF-stem cells of cashmere goats. As shown in Figure 2C, the si-circZNF638-1 and si-circ ZNF638-2 was more efficient in knockdown of m^6^A-circRNA-ZNF638 compared with that of si-circ ZNF638-3 (Figure 2C). Therefore, the si-circZNF638-1 and si-circ ZNF638-2 were selected and utilized in further experiments. As shown in Figure 2D, the si-circZNF638-1/-2-mediated knockdown of m6A-circRNA-ZNF638 resulted in a significant decrease in the expression of the analyzed indicator genes upon induced activation of SHF-stem cells in comparison to those of the control group (si-control) (Figure 2D). Thus, it can be inferred that m^6^A-circRNA-ZNF638 may be essentially implicated in contributing the induced activation of SHF-stem cells in cashmere goats through certain unknown mechanisms.

Recently, several studies showed that circRNAs were essentially implicated in the differentiation of SHF-stem cells into hair follicle lineage in cashmere goats through loss-of-function experiments [13–15], but little information is available about the functional role of m6A modified circRNAs in SHF-stem cells of cashmere goats. Also, it is unclear how circRNAs epigenetically modulate the induced activation and cell-fate decisions of SHF-stem cells of cashmere goats. Interestingly, here, we verified the m^6^A modification of circRNA-ZNF638 in SHF-stem cells of cashmere goats by performing a methylated RNA immunoprecipitation (Me-RIP) assay (Figure 2E), which suggests that m^6^A-circRNA-ZNF638 may play functional roles in contributing to induced activation of SHF-stem cells through m^6^A modification-dependent pattern.

### The m^6^A modification of circRNA-ZNF638 is implicated in its contributing to induced activation of SHF-stem cells

**Figure 3:**
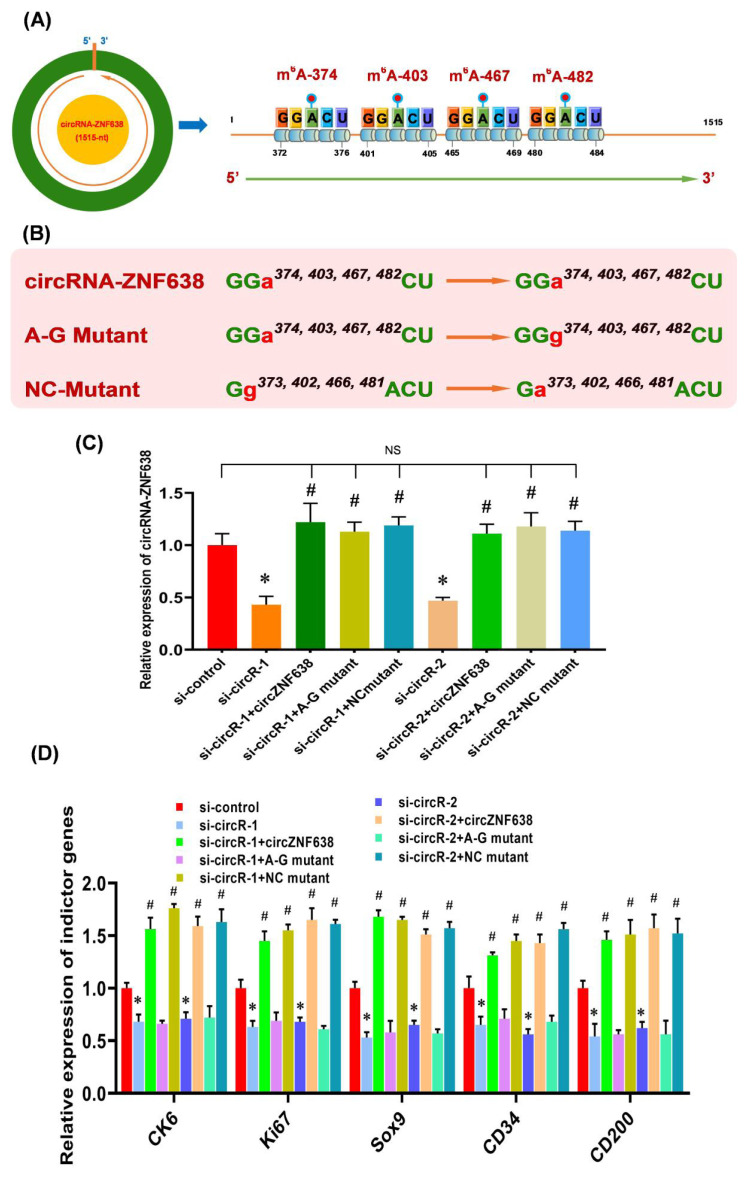
The m^6^A modification of circRNA-ZNF638 is implicated in its contributing to induced activation of SHF-stem cells. (A) Diagram of m^6^A sites within circRNA-ZNF638 sequences of cashmere goat SHFs including m^6^A-374, m^6^A-403, m^6^A-467, and m^6^A-482 (Hui et al [2]). (B) The construction strategies of mutated m^6^A sites for circRNA-ZNF638. circRNA-ZNF638 = wild type of circRNA-ZNF638 (WT), A-G Mutant = m^6^A site mutant of circRNA-ZNF638 (MUT), and NC mutant = negative control mutant. (C) The effects of m^6^A site mutation on circRNA-ZNF638 expression in circRNA-ZNF638 knockdown SHF-stem cells. (D) The effects of m^6^A site mutation on the expression indictor genes in circRNA-ZNF638 knockdown SHF-stem cells. circRNA-ZNF638, circRNA-zinc finger protein 638; SHF, secondary hair follicle. The asterisk (*) stands for a significant difference compared with ‘si-control’ (p<0.05), and the hash mark (#) stands for a significant difference compared with ‘si-circR-1’ or ‘si-circR-2’ (p<0.05). ‘NS’ stands for no significant difference among the different treated groups (p>0.05).

Recent studies have suggested that the m^6^A modifications are heavily implicated in the functional exertion of m^6^A-circRNAs [11,16]. This promotes us ask whether the observed functional role of m^6^A-circRNA-ZNF638 in contributing the induced activation of SHF-stem cells of cashmere goats was achieved through m^6^A-dependent pattern. As described in our previous publication [2], four m^6^A sites were revealed within circRNA-ZNF638 sequence, and they were named as m^6^A-374, m^6^A −403, m^6^A −467, and m^6^A −482, respectively (Figure 3A). Therefore, we constructed the expression vectors of circRNA-ZNF638 (wild type, WT) and its mutant expression vectors (mutant type, MUT), in which the adenine residues embedded within the m^6^A motifs (GGACU) were replaced with guanine residues (A-G mutation) (Figure 3B). We also constructed the mutants of circRNA-ZNF638 in which the four guanine residues (G^373^, G^402^, G^466^, and G^481^) within the m6A motifs (GGACU) were replaced with unmethylated adenine residues (G-A mutation) as control mutant (NC mutant) (Figure 3B) as reported by Yang and colleagues [26].

To evaluate the relationship between m^6^A modification and the functional role of circRNA-ZNF638 in SHF-stem cells, we overexpressed circRNA-ZNF638 (WT), the m^6^A-deficient A-G mutant (MUT) or the m^6^A-decorated mutant (NC mutant) with equal amounts in circRNA-ZNF638-knockdown SHF-stem cells and used them *in vitro* induced activation assays. We found that the mutations were not implicated in expression levels of circRNA-ZNF638, as there was no significant difference in circRNA-ZNF638 expression between the wild type- and mutant-transfected SHF-cells (Figure 3C). On the other hand, we also noted that the expression level of circRNA-ZNF638 was restored in circRNA-ZNF638-knockdown SHF-stem cells through the introduction of either wild type- circRNA-ZNF638 or its mutants (Figure 3C).

Next, we analyzed the expression changes of the indictor genes upon induced activation of SHF-stem cells in the transfected cells. As observed from Figure 3D, the ‘si-circR-1/2+circRNA-ZNF638’ cell lines, not the ‘si-circR-1/2+A-G mutant’ cells, exhibited a significantly higher expression of the analyzed indictor genes, compared with the si-circR-1/2 cell lines (Figure 3D). Notably, there is no significant difference in circRNA-ZNF638 expression levels between ‘si-circR-1/2+circRNA-ZNF638’ and ‘si-circR-1/2+A-G mutant’ cells (Figure 3C). Thus, we can rule out the possibility that the observed expression changes of the analyzed indictor genes between two cells types (si-circR-1/2+circRNA-ZNF638’ and ‘si-circR-1/2+A-G mutant’) were related with the circRNA-ZNF638 expression abundance. On the other hand, as expected, the NC mutant (m^6^A-decorated mutant) restored the indictor expression level of circRNA-ZNF638-deficient cells (Figure 3D). Taken together, these results suggest that the m^6^A modifications are necessary for circRNA-ZNF638 in contributing to the induced activation of SHF-stem cells in cashmere goats. A highly similar functional mechanism was also reported in mouse embryonic stem cells (mESCs), where the internal m^6^A modification was verified to positively participate in the functional role of linc1281 in the differentiation potential of mESCs [26].

### M^6^A-circRNA-ZNF638 functions as miR-361-5p sponge and may negatively regulate its expression

**Figure 4:**
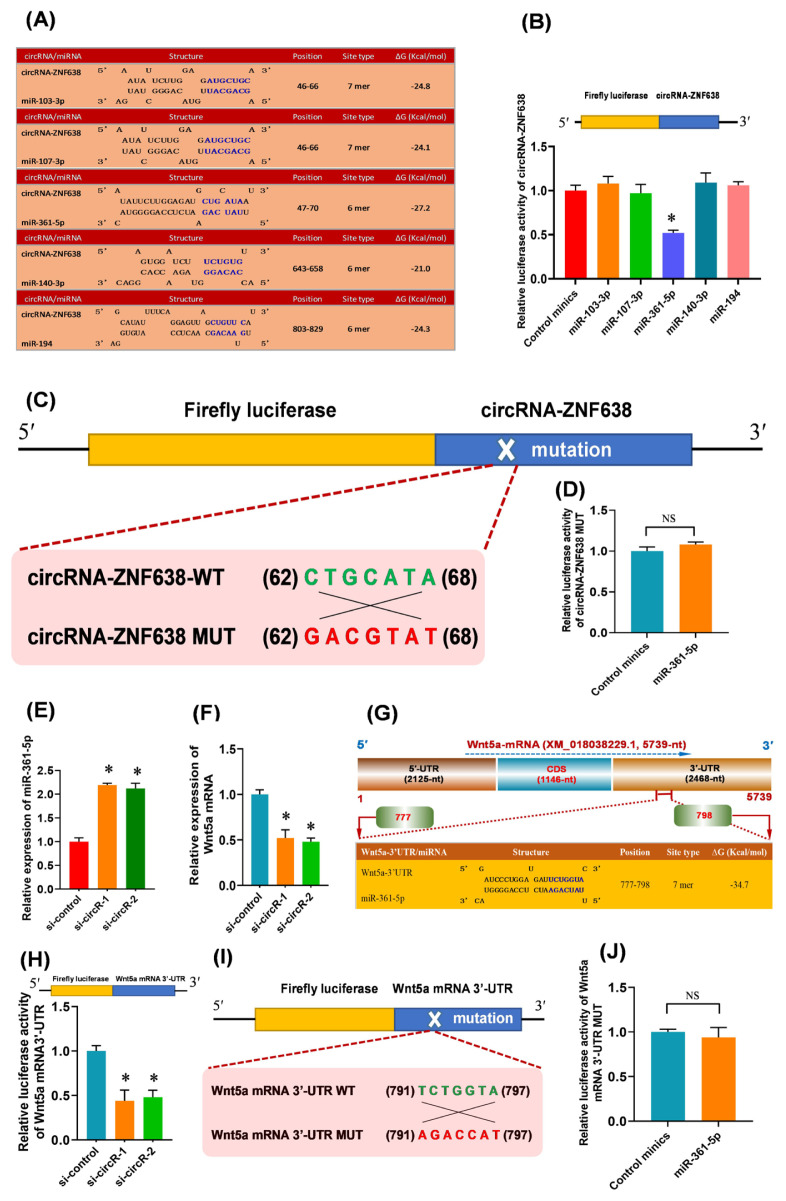
The m^6^A-circRNA-ZNF638 functions as miR-361-5p sponge to enhance the Wnt5a expression SHF-stem cells. (A) The prediction of the binding sites of target miRNAs within m^6^A-circRNA-ZNF638 including miR-103-3p, miR-107-3p, miR-261-5, miR-140-3p, and miR-194. (B) Relative luciferase activities of reporters containing circRNA-ZNF638 in SHF-stem cells 48 h after co-transfection with the indicted different miRNAs. (C) The construction strategies of circRNA-ZNF638 mutant (MUT) for the miR-361-5p binding sites within circRNA-ZNF638. (D) Relative luciferase activities of reporters containing circRNA-ZNF638 mutant (MUT) in SHF-stem cells 48 h after co-transfection with the miR-361-5p or control minics. (E) Expression analysis of miR-361-5p in circRNA-ZNF638 knockdown SHF-stem cells. (F) Expression analysis of Wnt5a mRNA in circRNA-ZNF638 knockdown SHF-stem cells. (G) An overall diagram of goat Wnt5a mRNA along with the prediction of potential binding sites of miR-361-5p on its mRNA 3′-UTR region. The nucleotide positions are indicated according to the goat KLF5 mRNA with accession no. XM_018056510 at NCBI (https://www.ncbi.nlm.nih.gov). (H) Relative luciferase activities of reporters containing Wnt5a 3′-UTR in circRNA-ZNF638 knockdown SHF-stem cells. (I) The construction strategies of Wnt5a mRNA-3′UTR mutant (MUT) for the miR-361-5p binding sites within Wnt5a mRNA-3′UTR. (J) Relative luciferase activities of reporters containing Wnt5a mRNA-3′UTR mutant (MUT) in SHF-stem cells 48 h after co-transfection with the miR-361-5p or control mimics. m^6^A-circRNA-ZNF638, *N*^6^-Methyladenosine modification-circRNA-zinc finger protein 638; SHF, secondary hair follicle; MUT, mutant. The asterisk “*” indicates significant difference (p<0.05). ‘NS’ stands for no significant difference among the different treated groups (p>0.05).

Previous studies have demonstrated that circRNAs can function as natural miRNA sponges to control gene expression through posttranscriptional regulation patterns [27]. Therefore, we screened the candidate target miRNAs of m^6^A-circRNA-ZNF638 via in-silico analysis. As shown in Figure 4A, five candidate miRNAs were found to have the potential binding sites within m^6^A-circRNA-ZNF638 sequence, including miR-103-3p, miR-107-3p, miR-361-5p, miR-140-3p, and miR-194 (Figure 4A). To determine which miRNAs were sequestered by m^6^A-circRNA-ZNF638, a luciferase reporter was constructed which contains full-length circRNA-ZNF638 in the 3′-UTR of luciferase gene. The reporters were co-transfected into SHF-stem cells with the single candidate miRNA mimics, respectively. As a result, in comparison to the control mimics, only the miR-361-5p led to a significantly decrease in the luciferase activities of the m^6^A-circRNA-ZNF638 reporters, but not for miR-103-3p, miR-107-3p, miR-140-3p, and miR-194 (Figure 4B). To further define the specifically binding of miR-361-5p to the predicted target site within m^6^A-circRNA-ZNF638, a mutant luciferase report (circRNA-ZNF-MUT) was constructed which harbors an antisense mismatch of 7-nt within seed target site of miR-361-5p (Figure 4C). When co-transfected with miR-361-5p, we found that the mutant reports were counteractive to the miR-361-5p-driven reporter suppression (Figure 4D). Thus, these findings suggest that m^6^A-circRNA-ZNF638 serves as a sponge via direct binding with miR-361-5p in SHF-stem cells of cashmere goats.

In addition, we found that the knockdown of m^6^A-circRNA-ZNF638 resulted in a significant increase of miR-361-5p expression in SHF-SCs (Figure 4E). However, neither overexpression nor knockdown of miR-361-5p had a significant effect on the expression of m^6^A-circRNA-ZNF638 in SHF-SCs of cashmere goats (data not shown). Therefore, we speculate that m^6^A-circRNA-ZNF638 may regulate the expression of miR-361-5p in SHF-stem cells. This regulatory mode is highly similar to that reported in investigation on the differentiation of cashmere goat SHF-stem cells into hair follicle lineage, where circRNA-0100 was revealed to directly bind with miR-153-3p, and negatively regulated its expression [15]. Also, in research on invasion and metastasis of colorectal cancer, Han and colleagues found that circLONP2 bound with miR-17, and negatively regulated its expression [28].

### M^6^A-circRNA-ZNF638 up-regulates the expression of Wnt5a in SHF-stem cells of cashmere goats through miR-361-5p mediated pathway

In previous studies, it was demonstrated that the Wnt signaling pathway was heavily implicated in the initiation of hair follicle development [29]. The Wnt5a, a member of Wnt family, has been shown to be critical for controlling the fate of hair follicle cells [29]. Moreover, it is believed that Wnt5a can promote the activation of human hair follicle stem cells leading to the onset of anagen stages [30]. These findings drove us to ask whether the Wnt5a may be implicated in the observed effect of m^6^A-circRNA-ZNF638 on the induced activation of SHF-stem cells through miR-361-5p mediated pathway. Therefore, we further investigated the expression change of Wnt5a in m^6^A-circRNA-ZNF638 knockdown SHF-stem cells. As shown in Figure 4F, after the knockdown of m^6^A-circRNA-ZNF638, the relative expression level of Wnt5a was significantly down-regulated in SHF-SCs of cashmere goats (Figure 4F). These results suggested that m^6^A-circRNA-ZNF638 may be positively related to the Wnt5a expression in the SHF-stem cells.

It was widely accepted that circRNA can act as miRNA sponge to reduce the active miRNAs, which disinhibits the expression of the miRNA target genes at the post-transcriptional level [31]. Here, we confirmed that m^6^A-circRNA-ZNF638 sequestered miR-361-5p (Figure 4B, 4C, and 4D), and positively regulated the expression of Wnt5a in SHF-stem cells (Figure 4F). These results raised a most likely mechanism that m6A-circRNA-ZNF638 may positively regulate the Wnt5a expression in SHF-stem cells via miR-361-5p mediated pathway. Thus, we performed an in-silico screen for potential binding sites of miR-361-5p within the 3′-UTR region of Wnt5a mRNA sequence. As shown in Figure 4G, a potential binding site of miR-361-5p was harbored within the 3′-UTR region of Wnt5a mRNA with a 7 mer binding type of seed region (Figure 4G). To confirm this interaction between miR-361-5p and the 3′-UTR region of Wnt5a mRNA, we constructed a luciferase reporter of 3′-UTR region of Wnt5a mRNA containing the potential binding site for miR-361-5p, and co-transfected the constructed reporter into the SHF-stem cells with si-RNAs of m^6^A-circRNA-ZNF638. As shown in Figure 4H, the si-circR-1/2 mediated knockdown of m^6^A-circRNA-ZNF638 (si-circR-1/2) in SHF stem cells significantly decreased the relative luciferase activity of Wnt5a mRNA 3′-UTR compared with siRNA control (si-control) (Figure 4H). In further order to verify the specifically binding of miR-361-5p to the predicted target site within the 3′-UTR region of Wnt5a mRNA, a mutant luciferase report (Wnt5a mRNA 3′-UTR-MUT) was constructed which harbors an antisense mismatch of 7-nt within seed target site of miR-361-5p (Figure 4I). When co-transfected into SHF-stem cells with miR-361-5p, we found that relative luciferase activity of mutant reports (Wnt5a mRNA 3′-UTR-MUT) had no significant change compared with that of miRNA control minics (Figure 4J). These results indicated the specifically target binding of miR-361-5p to 3′-UTR region of Wnt5a mRNA.

In fact, functionally, it was demonstrated that circRNAs could play roles in cells through multiple pathways and mechanisms, such as, sponging miRNAs [27], regulating transcription of host gene [32–35], controlling RNA transport [36], and serving as template for protein translation [11]. Here, we showed that m^6^A-circRNA-ZNF638 sponged miR-361-5p to upregulate the expression of Wnt5a in SHF-stem cells of cashmere goats. However, the other functional roles of m^6^A-circRNA-ZNF638 in SHF-stem cells of cashmere goats should be further investigated, such as its potential function of regulating ZNF638 transcription, and encoding a protein which may have essentially significant to the physiological function of SHF-stem cells in cashmere goats.

### The m^6^A modification is necessary for the circRNA-ZNF638-mediated regulatory effects through miR-361-5p/Wnt5a axis

**Figure 5:**
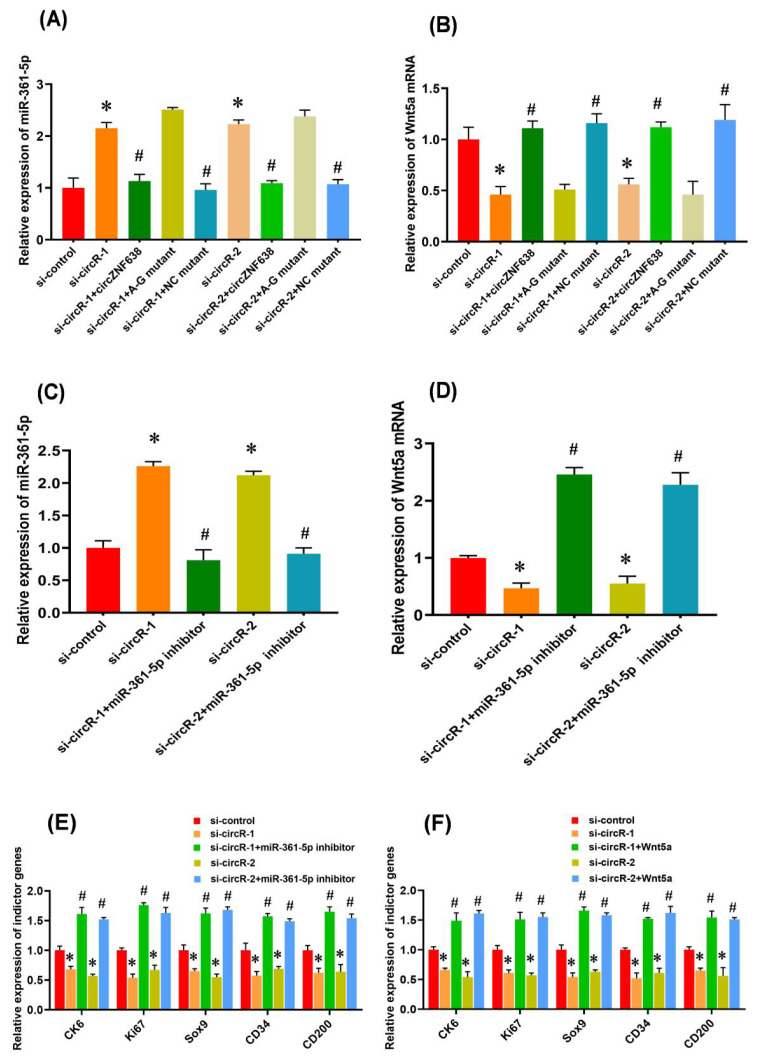
The m^6^A modification is necessary for circRNA-ZNF638 function through miR-361-5p/Wnt5a pathway that restored the induced activation of SHF stem cells with m^6^A-circRNA-ZNF638-deficient. (A) The effects of m^6^A site mutation on miR-361-5p expression in circRNA-ZNF638 knockdown SHF-stem cells. (B) The effects of m^6^A site mutation on Wnt5a mRNA expression in circRNA-ZNF638 knockdown SHF-stem cells. (C) The miR-361-5p inhibitor led to a significant decrease of its expression in circRNA-ZNF638 knockdown SHF-stem cells. (D) The miR-361-5p inhibitor led to a significant increase of Wnt5a mRNA expression in circRNA-ZNF638 knockdown SHF-stem cells. (E) The miR-361-5p inhibitor led to significantly increased expression of the indictor genes in circRNA-ZNF638 knockdown SHF-stem cells. (F) Overexpression of Wnt5a gene led to significantly increased expression of the indictor genes in circRNA-ZNF638 knockdown SHF-stem cells. m^6^A-circRNA-ZNF638, *N*^6^-Methyladenosine modification-circRNA-zinc finger protein 638; SHF, secondary hair follicle. The asterisk (*) stands for a significant difference compared with ‘si-control’ (p<0.05), and the hash mark (#) stands for a significant difference compared with ‘si-circR-1’ or ‘si-circR-2’ (p<0.05).

There is evidence that m^6^A modifications within RNA molecules are significantly implicated in various cellular biological processes, such as miRNA biogenesis initiation [35], pre-mRNA splicing [36], and circRNA translation initiation [11]. Although, the functional significance of m^6^A modification within circRNAs still needs to be clarified, an m^6^A -dependent model was reported on the interaction between lncRNA and miRNA in which m6A modification of linc1281 was found to be required for its directly binding with let-7 family miRNAs [26]. This promotes us ask whether m^6^A modification of circRNA-ZNF638 is necessary for its regulatory effect on the induced activation of SHF stem cells through miR-361-5p/Wnt5a axis. To define this hypothesis, we first tested the expression level of miR-361-5p in ‘siRNA-circR-1/2+circRNA-ZNF638’, ‘siRNA-circR-1/2+ circRNA-A-G mutant’, and ‘siRNA-circR-1/2+NC mutant’ SHF-stem cells. We noted that ‘siRNA-circR-1/2+circRNA-ZNF638’, and ‘siRNA-circR-1/2+NC mutant’ cells exhibited decreased miR-361-5p expression compared with ‘si-circR-1/2’ cells (Figure 5A). However, the ‘siRNA-circR-1/2+A-G mutant’ cells still exhibited high miR-361-5p expression levels (Figure 5A), despite there is no significant difference in circRNA-ZNF638 transcript levels among these different treated cell types (Figure 3C). We further examined the expression level of Wnt5a mRNA in ‘siRNA-circR-1/2+ circRNA-ZNF638’, ‘siRNA-circR-1/2+circRNA-A-G mutant’, and ‘siRNA-circR-1/2+NC mutant’ SHF-stem cells. We found that the ‘siRNA-circR-1/2+A-G mutant’ cells exhibited decreased Wnt5a mRNA expression compared with ‘si-circR-1/2’ cells (Figure 5B). However, the ‘siRNA-circR-1/2+circRNA-ZNF638’, and ‘siRNA-circR-1/2+NC mutant’ cells still displayed high Wnt5a mRNA levels (Figure 5B). Taken together, it appears to become apparent that the m^6^A modification is necessary for the circRNA-ZNF638-mediated regulatory effects through miR-361-5p/Wnt5a axis.

In fact, it is widely accepted that the binding between circRNAs (or lncRNAs) and miRNAs is usually driven through a sequence-based complementary base pairing model in which whether regulators mediate this binding is still unclear [14,26]. Here, we showed that m^6^A positively mediated the binding between m^6^A-circRNA-ZNF638 and miR-361-5p, which is revealed by m^6^A-deficient A-G mutant of circRNA-ZNF638 that abolished the binding of circRNA-ZNF638 with miR-361-5p (Figure 5A and 5B). On the other hand, previous investigations on m^6^A peak of mRNA and miRNA binding sites have suggested a possible mechanism via which m^6^A might cooperate or compete with miRNAs to ultimately regulate the expression of target mRNAs [37]. In this study, we suggested a model that circRNAs harbor both m^6^A peaks and miRNA binding sites in which m^6^A modification cooperate with miRNA to drive the circRNA-mediated ceRNA regulation, thereby contributing to the induced activation of SHF-stem cells of cashmere goats. Although it remains unclear that whether m^6^A modification alters the local structure of circRNA-ZNF638 as reported in a previous publication [38] thereby driving the binding with miR-361-5p, our results provide significant insights into the m^6^A mediated regulatory model of circRNA in SHF-stem cells of cashmere goats.

### The miR-361-5p/Wnt5a axis restores the induced activation of SHF stem cells with m^6^A-circRNA-ZNF638-deficient

And then, we asked whether the miR-361-5p/Wnt5a axis is responsible for the m^6^A-circRNA-ZNF638-mediated regulation on the induced activation of SHF-stem cells. For this purpose, miR-361-5p inhibitor was transfected into m6A-circRNA-ZNF638 knockdown cells. As expected, the miR-361-5p expression was significantly decreased by its inhibitor in circRNA-ZNF638 knockdown SHF-stem cells (Figure 5C). Coupled with the observation, Wnt5a mRNA level was significantly increased by the miR-361-5p inhibitor in circRNA-ZNF638 knockdown SHF-stem cells (Figure 5D). Interestingly, we found that concomitant inhibition of miR-361-5p was sufficient to rescue the impaired inducted activation of SHF-stem cells after m^6^A-circRNA-ZNF638 knockdown, which can be determined by the significant up-regulation of the indicator genes on induced activation of SHF-stem cells (Figure 5E).

On the other hand, since Wnt5a mRNA was revealed to bind directly with miR-361-5p (Figure 4G-4J), and showed decreased levels upon m^6^A-circRNA-ZNF638 knockdown (Figure 5B). We also examined whether Wnt5a contributed to the underlying mechanism of the m^6^A-circRNA-ZNF638/miR-361-5p model. We overexpressed Wnt5a in SHF-stem cells with m^6^A-circRNA-ZNF638 knockdown. As shown in Figure 5F, the overexpression of Wnt5a restored the inducted activation of SHF-stem cells caused by m^6^A-circRNA-ZNF638 deficiency, which can be determined by the significant up-regulation of the indicator genes on induced activation of SHF-stem cells (Figure 5F).

**Figure 6:**
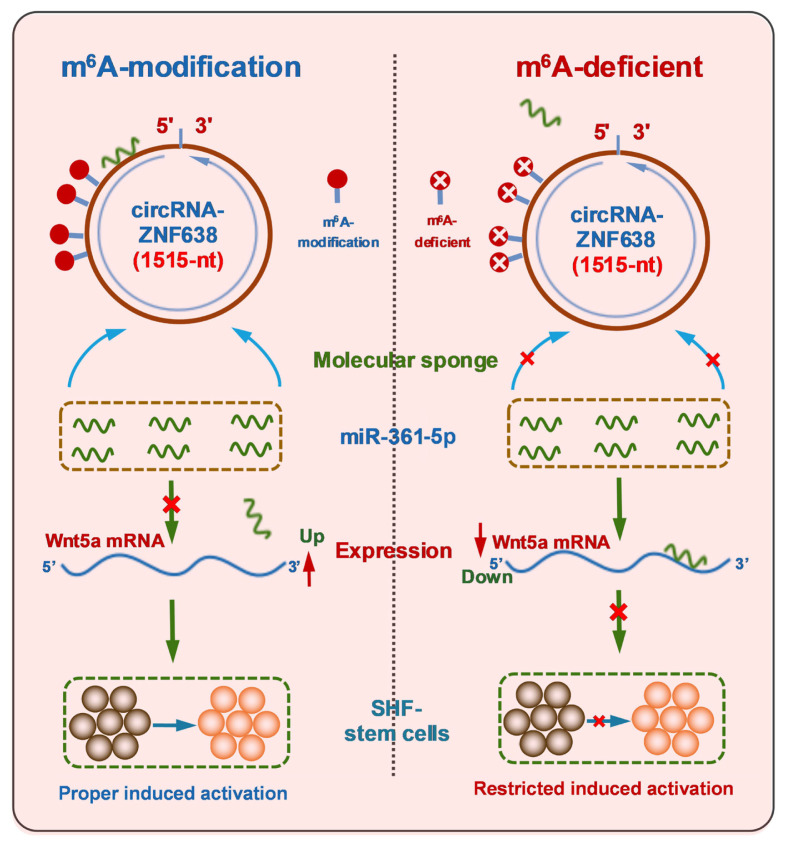
A schematic representation of functional mechanism of m^6^A-circRNA-ZNF638 in contributing to the induced activation of SHF-stem cells in cashmere goats in which m^6^A modification within circRNA-ZNF638 is required for mediating the miR-361-5p/Wnt5a pathway. m^6^A-circRNA-ZNF638, *N*^6^-Methyladenosine modification-circRNA-zinc finger protein 638; SHF, secondary hair follicle.

Previously, although it was thought that Wnt5a, as a member of non-canonical Wnt family, could inhibit the canonical Wnt/β-catenin signaling that was essentially involved in the initiation of hair follicle development [29], and it is not yet known whether non-canonical Wnt5a is directly implicated in the induced activation event of SHF-stem cells of cashmere goats. However, it was reported that Wnt5a also could activate canonical Wnt/β-catenin signaling during mouse embryonic development [39]. Moreover, compared with catagen and the telogen, Wnt5a exhibited the highest levels at the anagen hair follicles with being prominently located in the matrix, precortex cells, inner root sheath, outer root sheath and the dermal papilla [29]. Also, it was found that overexpression of Wnt5a in DPCs led to a significant decrease in expression of the related genes involved in maintaining cell quiescent state [40]. In addition, Wnt5a can serve as both SHH signaling target and Notch/CSL signaling mediator in the morphogenesis and differentiation of hair follicles, respectively [41]. Here, we found that m^6^A modification of circRNA-ZNF638 of cashmere goats is required for the induced activation of SHF stem cells through miR-361-5p/Wnt5a axis (Figure 6). Nevertheless, it is worth noting that, here, we conducted the experiment in SHF-stem cells *in vitro*. Therefore, the revealed functional mechanism of m^6^A-circRNA-ZNF638 above should be further confirmed in SHF-stem cells *in vivo*.

## CONCLUSION

The m^6^A modification of circRNA-ZNF638 contributes the proper induced activation of SHF-stem cells in cashmere goats, and this important functional role relies upon m^6^A modification on circRNA-ZNF638 in which m^6^A-modified circRNA-ZNF638 sequesters miR-361-5p to heighten Wnt5a expression.

## Figures and Tables

**Table 1:** Detail of polymerase chain reaction primers utilized in this study and their polymerase chain reaction reaction assay

Gene name	Reference	Sequence (5′-3′)	Primer length (nt)	Amplicon size (bp)	Annealing temperature (°C)
*CircRNA-ZNF638 (Divergent primers)*	Hui et al [2]	F:TTTGCAATGCCCAGGTTCAA,	20	250	55
		R:CTGACATCCCCATTCGGTCT	20		
*CK6*	XM_018047719.1 in GenBank	F:AGTTTGCCTCCTTCATCG,	18	111	53
		R:GGTTCTGCTTCACGGTCTT	19		
*Ki67*	XM_005698601.3 in GenBank	F:AGGAAGTAGCCAGACTGAGGG,	21	143	56
		R:GCATCGTGGTTTGCTGTGAA	20		
*Sox9*	XM_018063905.1 in GenBank	F:GGTGCTCAAGGGCTACGACTGG	22	162	60
		R:GCGTTGTGCAGGTGCGGGTA	20		
*CD34*	XM_018060788.1 in GenBank	F:GAAGATGTCAGCAGCCACCAG	21	112	56
		R:GGCGGTTCATCAGGAAATAGCAC	23		
*CD200*	XM_005674916.3 in GenBank	F:TTGGAAGATGAGGCGTGTTA	20	156	54
		R:AGCATTGGCAGAGCAAGTGA	20		
*GAPDH*	XM_005680968.3	F:TGAACCACGAGAAGTATAACAACA	24	125	53
		R:GGTCATAAGTCCCTCCACGAT	21		
*CircRNA-ZNF638 (Me-RIP-qPCR)*	This study	F: TCATTGTCTGATTAACTGTCTGGCT	25	167	53
		R: GTTTCGACAGATGGACTTCCC	21		
*miR-361-5p*	MIMAT0036171 in miRNAsong	F: GCTTATCAGAATCTCCAGGGGTAC	24	NA	60
*U6*	Han et al [20]	F: CGCTTCGGCAGCACATATAC	20	NA	55
		R:AAATATGGAACGCTTCACGA	20		
*Wnt5a*	XM_018038229.1	F: ACATCACTTGGCGAATGGACG	21	118	57
		R: CTGGGAGCAGTTCTCGGGACA	21		

*CircRNA-ZNF638*, circRNA-zinc finger protein 638; *CK6*, cytokeratin 6; *Ki67*, marker of proliferation Ki-67; *Sox9*, SRY-box transcription factor 9; *CD34*, CD34 molecule; *CD200*, CD200 molecule; *GAPDH*, glyceraldehyde-3-phosphate dehydrogenase; *U6*, U6 small nuclear RNA; *Wnt5a*, Wnt family member 5A; NA, not available.
